# Incidental Breast Hemangioma on Breast MRI: A Case Report

**DOI:** 10.7759/cureus.57903

**Published:** 2024-04-09

**Authors:** Ashley Bancroft, John Santa Cruz, Kaitlyn Levett, Quan D Nguyen

**Affiliations:** 1 Radiology, Baylor College of Medicine, Houston, USA; 2 Pathology and Immunology, Baylor College of Medicine, Houston, USA

**Keywords:** breast ultrasound, mri breast, screening mammogram, rare vascular tumor, benign vascular tumor, breast hemangioma

## Abstract

Vascular tumors of the breast are rare, but benign hemangiomas are the most common type. Capillary hemangiomas are a subset of benign vascular tumors that involve smaller vessel sizes. They are difficult to diagnose with mammography and ultrasound, as they lack pathognomonic features and are frequently not seen. MRI is the most sensitive imaging tool. The lesions appear similar to angiosarcoma or ductal carcinoma in situ on imaging, which further complicates the diagnosis. A biopsy of the lesions is required for a definitive diagnosis. In this report, a 49-year-old female with newly diagnosed breast cancer is incidentally found to have a capillary hemangioma on staging breast MRI that was confirmed with a biopsy and excised along with the primary breast cancer with a partial mastectomy. The imaging findings of breast hemangioma on mammography, ultrasound, and MRI are also reviewed and described in this report.

## Introduction

In the breast, hemangiomas are rare masses comprised of small-caliber, benign blood vessels categorized as either capillary or cavernous based on the vessel size involved [1,2]. These are often found incidentally on imaging or on pathology for mastectomy. When discovered, MRI is the best available imaging modality for evaluation [3]; however, the lack of pathognomonic imaging characteristics makes diagnosis challenging without tissue sampling. In the current literature, there is no direct evidence to support these lesions having potential for malignant transformation; however, angiosarcoma can show benign pathology such as hemangioma on a core needle biopsy, so the clinical scenario is very important in terms of management [4-6].

In our case, the hemangioma was found on a staging MRI for a recently diagnosed ipsilateral breast cancer, so management included biopsy and eventual excision with partial mastectomy.

## Case presentation

**Figure 1:**
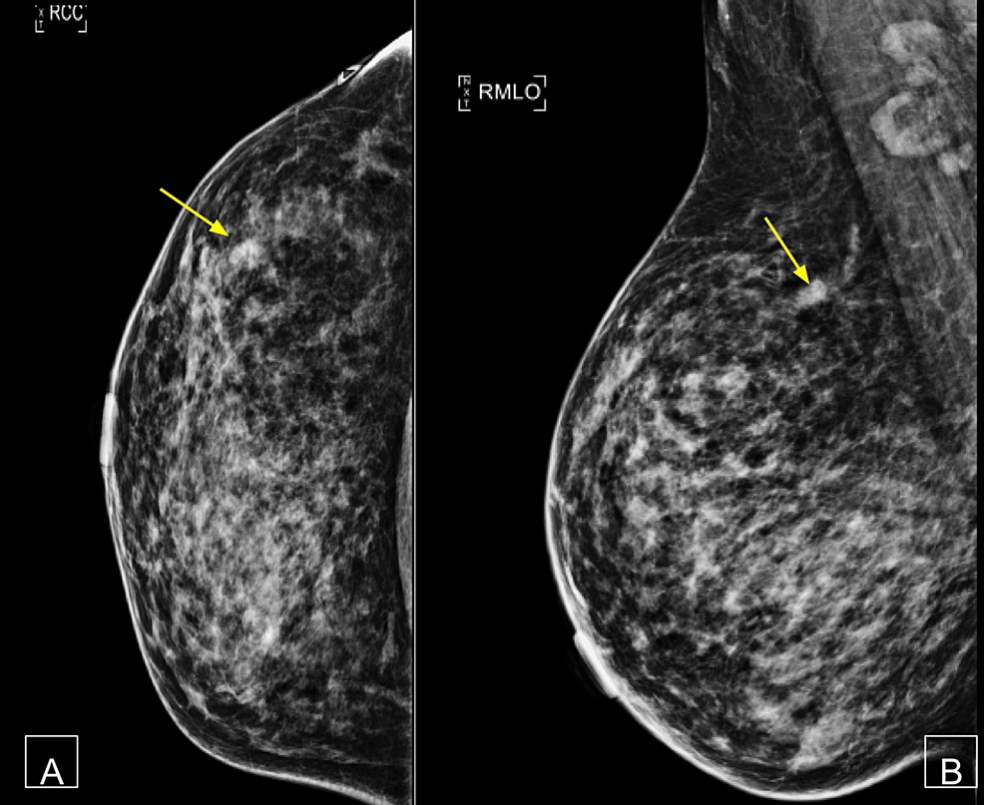
Suspicious right breast mass on mammography (A) MLO and (B) CC views demonstrate a 1.7 cm irregular, noncalcified right breast mass (arrows) in the 10:00 position, suspicious of malignancy. CC, craniocaudal; MLO, mediolateral oblique

**Figure 2:**
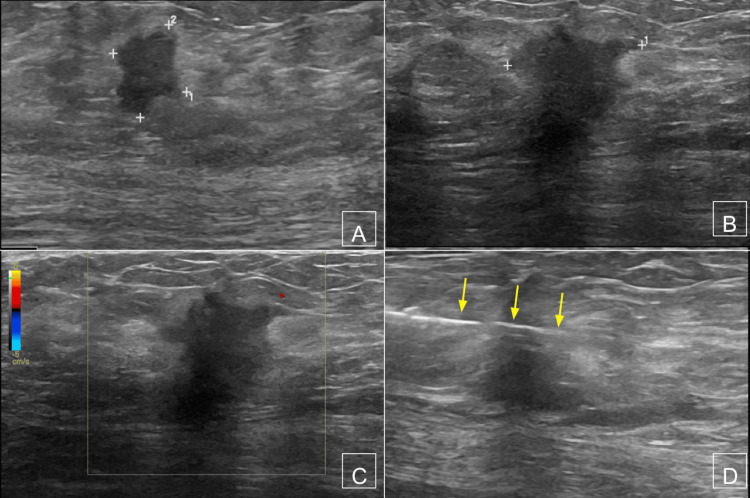
Suspicious right breast mass on sonography (A) Transverse and (B) longitudinal grayscale and (C) sagittal color Doppler images demonstrate 1.9 × 1 × 1.8 cm of spiculated mass in the right breast, corresponding to mammographic mass. An ultrasound-guided biopsy (D) revealed invasive ductal carcinoma (arrows = needle).

**Figure 3:**
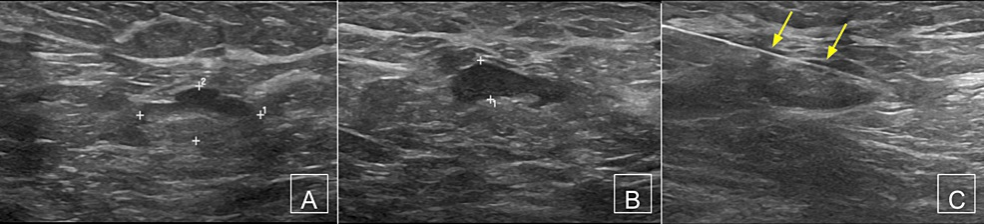
Suspicious axillary lymph node on sonography (A and B) Long-axis grayscale images through the 1.6 × 0.7 × 1.5 cm axillary lymph node demonstrated suspicious focal cortical thickening, prompting a biopsy. (C) An ultrasound-guided biopsy revealed metastatic ductal carcinoma (arrows = needle).

A 49-year-old woman presented to the breast imaging clinic after a referral from her primary care physician for a patient who had felt a lump in her right breast for one month. The patient had no relevant past medical history. Family history was significant for a sister diagnosed with breast cancer at age 51. The diagnostic mammogram showed a 1.7 cm irregular right breast mass, with subsequent ultrasound revealing a 1.7 cm irregular, spiculated mass at 10 o’clock posterior depth and a right axillary lymph node with cortical thickening (Figures 1-3). An ultrasound-guided biopsy of the mass and node was performed without complications, and pathology revealed infiltrating ductal carcinoma with involvement of the axillary node.

**Figure 4:**
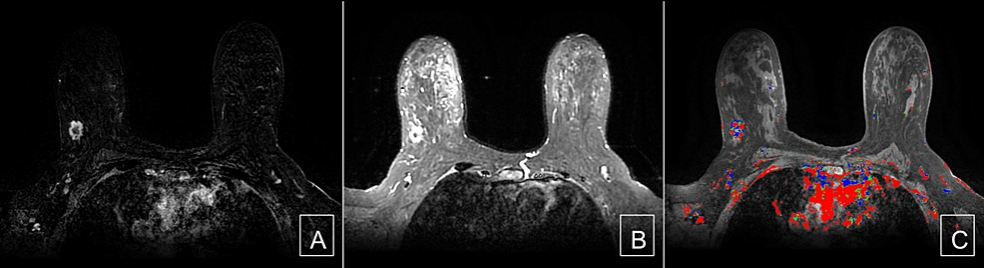
Cancer: subtracted image, T2 image, and kinetics image (A) A fat-saturated subtracted T1 weighted post-contrast image demonstrates irregular mass with irregular margins with heterogeneous internal enhancement. (B) A fat-saturated T2-weighted image demonstrates a high signal within the invasive ductal carcinoma. (C) A fat-saturated T1-weighted post-contrast with kinetic overlay demonstrates predominantly rapid washout kinetics.

**Figure 5:**
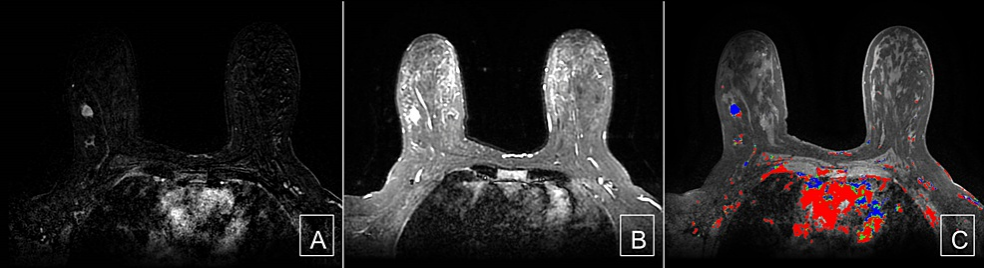
Hemangioma: subtracted image, T2 image, and kinetics image (A) A fat-saturated subtracted T1 weighted post-contrast image demonstrates irregular mass with irregular margins with heterogeneous internal enhancement. (B) A fat-saturated T2-weighted image demonstrates a high signal within the capillary hemangioma. (C) A fat-saturated T1-weighted post-contrast with kinetic overlay demonstrates predominantly persistent washout kinetics.

**Figure 6:**
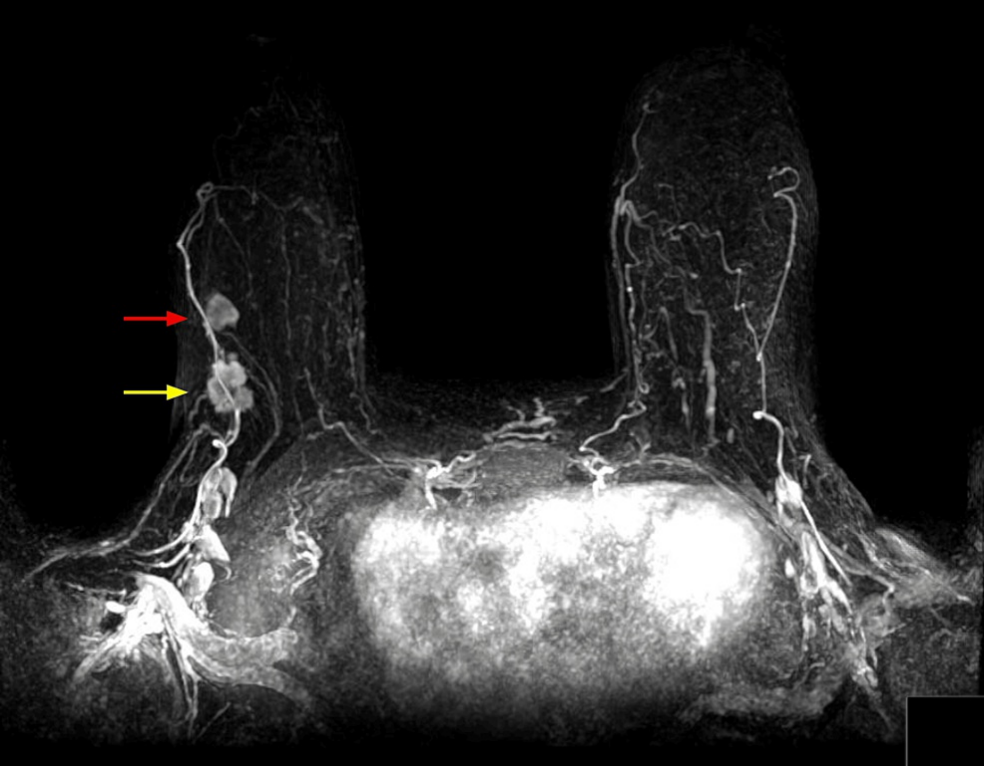
Maximal intensity projection Maximal intensity projection reconstruction demonstrating both the right breast cancer (yellow arrow) at 10 o’clock at posterior depth and the right breast capillary hemangioma (red arrow) at 10 o’clock at middle depth.

**Figure 7:**
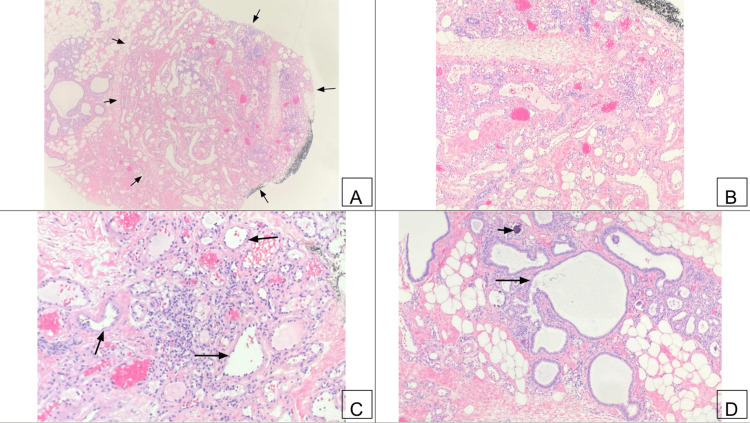
Breast hemangioma on histology (H&E) (A) Histology of the hemangioma (arrows) shows well-demarcated proliferation of small, thin-walled blood vessels within the interlobular stroma (20× magnification). (B) Endothelial cells of the vascular proliferation range from flattened to plump (40× magnification). Cytologic atypia and mitoses are absent. (C) Magnification shows proliferation of thin-walled blood vessels of varying sizes, with smaller capillaries (arrows) and plump endothelial lining more toward the center of the lesion (100× magnification). (D) A terminal duct lobular unit (arrow) and epithelial cells displaying columnar cell change without any cytologic atypia (100× magnification). Magenta-colored microcalcifications (short arrow) are present in the luminal spaces associated with epithelial columnar cell change.

A breast MRI was subsequently performed to evaluate the extent of the disease. The malignancy was visualized on MRI as an irregular mass with irregular margins and heterogeneous internal enhancement, demonstrating rapid washout kinetics (Figure 4). Anterior to the biopsy-proven malignancy in the right breast, there was a 1.3 cm oval mass with circumscribed margins and heterogeneous internal enhancement demonstrating persistent washout kinetics, which did not have a clear mammographic correlate retrospectively (Figures 5, 6). The lesion was scored as BI-RADS 4, and targeted ultrasound could not find a sonographic correlate. An MRI-guided core needle biopsy was thus performed, with pathology showing capillary hemangioma with columnar cell change and hyperplasia associated with microcalcifications (Figure 7). The radiology and pathology were determined to be concordant. The patient underwent a partial mastectomy and axillary lymph node dissection for the infiltrating ductal carcinoma, and the entire hemangioma was included in the resection specimen, further showing benign capillary hemangioma.

## Discussion

Hemangiomas result from the malformation of mature blood vessels [7]. While there are multiple subtypes, such as cavernous and capillary, the distinction is clinically insignificant. The tumors can occur in the breast of any age group, with an increased incidence in women exposed to hormonal therapy [8]. Hemangiomas are often incidentally found on lumpectomy, mastectomy, or postmortem exams. A physical exam is inconsistent and variable; some hemangiomas manifest as discolored, superficial masses, while others are not appreciated on the physical exam at all. Imaging findings are also just as variable and are often not visualized at all.

When they are visualized, mammography typically reveals circumscribed, superficial oval lesions. The majority of hemangiomas are smaller than 1 cm and outside of the fibroglandular tissue, located in the subdermis or subcutaneous tissue. Appearance is nondescript, often appearing indistinct from complicated cysts or other benign nonvascular masses. Hemangiomas generally have more consistent features on ultrasound compared to mammography [9]. Most of these tumors are hypoechoic; however, there is variability in the echotexture. Furthermore, most hemangiomas appear on sonography as circumscribed masses that occasionally have microlobulated margins [6]. Color Doppler may be useful in determining if the lesion is benign, as angiosarcomas are hypervascular and hemangiomas tend to be hypovascular [10]. However, sonography is still not definitively diagnostic. MRI is also not a reliable imaging tool for identifying and diagnosing hemangiomas, but it is the most sensitive for discovering their presence. For instance, in our case, the patient’s hemangioma was not seen on mammography or targeted ultrasound and was only evident on an MRI. Generally speaking, hemangiomas are circumscribed masses with diffuse enhancement with varying intensity on both T1 and T2 weighted sequences. There are reports of early contrast enhancement causing suspicion of malignancy [11]. Our case demonstrated enhancement with persistent kinetics and scattered areas of washout, which pushed the patient toward biopsy. In summary, hemangiomas lack pathognomonic features across all imaging modalities [12].

Breast hemangiomas are the benign counterpart to the malignant malformation of blood vessels, angiosarcoma. The two types of vascular tumors are difficult to distinguish on imaging alone because they lack pathognomonic features. Hemangiomas can also be imaged similarly to ductal carcinoma in situ on mammography when calcifications are present, most commonly phlebolith [13,14]. For these reasons, suspicious lesions should always be biopsied to delineate benign from malignant tumors. Biopsies of the lesions are occasionally bloody due to the vascular nature of the tumor [15].

There is ongoing debate about the necessity of surgical excision for further evaluation of hemangiomas. Traditionally, all hemangiomas were surgically excised to exclude any component of well-differentiated angiosarcoma. It has been proposed that there is potential for malignant transformation of benign hemangiomas into angiosarcomas, although there is no convincing evidence in the literature to support this currently. However, angiosarcoma can show benign pathology such as hemangioma on a core needle biopsy, which makes clinical suspicion of angiosarcoma vital to help guide management, such as if the lesion is rapidly enlarging or symptomatic [5]. For benign hemangiomas, the clinical course is widely unremarkable without complete excision, so the narrative has shifted away from this additional procedure. Per current recommendations, only tumors with biopsies concerning signs of malignancy, such as atypical features or radiopathologic disconcordance, need to be excised. Asymptomatic hemangiomas with radiopathologic concordance can be monitored with follow-up imaging [16,17].

## Conclusions

This case discusses a 49-year-old woman with diagnosed breast carcinoma who was incidentally found to have a breast capillary hemangioma on a staging MRI. Capillary hemangiomas are a type of benign vascular tumor that is difficult to diagnose with imaging as they have varying presentations across modalities. However, MRI is the most reliable and sensitive tool. A biopsy is necessary for a definitive diagnosis and to ensure radiopathologic concordance in order to rule out malignant imitators. Further management with surgical excision is decided on a case-by-case basis.
